# Baseline situational analysis in Bangladesh, Jordan, Paraguay, the Philippines, Ukraine, and Zimbabwe for the WHO Special Initiative for Mental Health: Universal Health Coverage for Mental Health

**DOI:** 10.1371/journal.pone.0265570

**Published:** 2022-03-22

**Authors:** Christopher G. Kemp, Tessa Concepcion, Helal Uddin Ahmed, Nazneen Anwar, Florence Baingana, Ian M. Bennett, Andrea Bruni, Dan Chisholm, Hania Dawani, Marcia Erazo, Saima Wazed Hossain, James January, Alisa Ladyk-Bryzghalova, Hasina Momotaz, Edmore Munongo, Renato Oliveira e Souza, Giovanni Sala, Alison Schafer, Oleksii Sukhovii, Luis Taboada, Mark Van Ommeren, Ann Vander Stoep, Jasmine Vergara, Chloe Waters, Devora Kestel, Pamela Y. Collins

**Affiliations:** 1 Department of International Health, Johns Hopkins University, Baltimore, MD, United States of America; 2 Department of Global Health, University of Washington, Seattle, WA, United States of America; 3 Child, Adolescent and Family Psychiatry, National Institute of Mental Health, Dhaka, Bangladesh; 4 Mental Health and Substance Use, WHO Regional Office for South East Asia, New Delhi, India; 5 UHC Communicable and Non Communicable Diseases Cluster, WHO Regional Office for Africa, Brazaville, Congo; 6 Department of Family Medicine, University of Washington, Seattle, WA, United States of America; 7 Department of Psychiatry and Behavioral Sciences, University of Washington, Seattle, WA, United States of America; 8 Iraq Country Office Team, World Health Organization, Baghdad, Iraq; 9 Division of NCDs and Promotion of Health through the Life-course, WHO Regional Office for Europe, Copenhagen, Denmark; 10 Jordan Country Office Team, World Health Organization, Amman, Jordan; 11 Non-communicable Diseases and Mental Health, PAHO/WHO Paraguay Country Office, Asunción, Paraguay; 12 National Advisory Committee for Autism and Neurodevelopmental Disabilities, Ministry of Health and Family Welfare, Dhaka, Bangladesh; 13 Shuchona Foundation, Dhaka, Bangladesh; 14 Barry University, Florida, FL, United States of America; 15 Department of Community Medicine, University of Zimbabwe College of Health sciences, Harare, Zimbabwe; 16 Mental Health Unit, WHO Country Office for Ukraine, Kyiv, Ukraine; 17 Non-communicable Diseases and Mental Health, World Health Organization, Bangladesh, Dhaka, Bangladesh; 18 UHC Communicable and Non Communicable Diseases Cluster, WHO country office Zimbabwe, Harare, Zimbabwe; 19 Mental Health and Substance Use Unit, PAHO/WHO, Washington, D.C., United States of America; 20 Department of Mental Health and Substance Use, World Health Organization, Geneva, Switzerland; 21 Unit of Community-based Mental Health Care, Mental Health in Primary Care and Rehabilitation, Center for Mental Health and Monitoring of Drugs and Alcohol of the Ministry of Health of Ukraine, Kyiv, Ukraine; 22 Mental Health Directorate, Ministry of Public Health and Social Welfare, Asunción, Paraguay; 23 Division of Programmes for Disease Control (DDC), World Health Organization Regional Office for the Western Pacific, Manila, Philippines; 24 Mental Health and Substance Use, World Health Organization, Philippines, Manila, Philippines; 25 Fred Hutchinson Cancer Research Center, Statistical Center for HIV/AIDS Research and Prevention, Seattle, Washington, United States of America; Columbia University College of Physicians and Surgeons, UNITED STATES

## Abstract

**Introduction:**

Mental, neurological and substance use conditions lead to tremendous suffering, yet globally access to effective care is limited. In line with the 13^th^ General Programme of Work (GPW 13), in 2019 the World Health Organization (WHO) launched the *WHO Special Initiative for Mental Health*: *Universal Health Coverage for Mental Health* to advance mental health policies, advocacy, and human rights and to scale up access to quality and affordable care for people living with mental health conditions. Six countries were selected as ‘early-adopter’ countries for the WHO Special Initiative for Mental Health in the initial phase. Our objective was to rapidly and comprehensively assess the strength of mental health systems in each country with the goal of informing national priority-setting at the outset of the Initiative.

**Methods:**

We used a modified version of the Program for Improving Mental Health Care (PRIME) situational analysis tool. We used a participatory process to document national demographic and population health characteristics; environmental, sociopolitical, and health-related threats; the status of mental health policies and plans; the prevalence of mental disorders and treatment coverage; and the availability of resources for mental health.

**Results:**

Each country had distinct needs, though several common themes emerged. Most were dealing with crises with serious implications for population mental health. None had sufficient mental health services to meet their needs. All aimed to decentralize and deinstitutionalize mental health services, to integrate mental health care into primary health care, and to devote more financial and human resources to mental health systems. All cited insufficient and inequitably distributed specialist human resources for mental health as a major impediment.

**Conclusions:**

This rapid assessment facilitated priority-setting for mental health system strengthening by national stakeholders. Next steps include convening design workshops in each country and initiating monitoring and evaluation procedures.

## Introduction

Mental, neurological and substance use conditions lead to tremendous suffering, disability, premature death, and social cost. Combined, they are the leading global causes of disability [1, 2]. Depression and anxiety disorders cost the global economy an estimated US$1 trillion per year [3]; suicides—a sequela of some mental disorders—account for 703,000 deaths annually [4]; perinatal mental disorders have intergenerational effects that can disturb a child’s growth, development, and future mental health [5, 6]; and people living with mental, neurological and substance use disorders face discrimination, social exclusion, and human rights abuses [7]. Mental disorders are also linked to communicable (e.g., HIV, COVID-19) and chronic, non-communicable diseases (e.g., cardiovascular disease) [8], often conferring risk for greater morbidity. The COVID-19 pandemic is illustrative: having a mental disorder is associated with greater risk of COVID-19 infection, hospitalization and mortality in some countries [9].

Mental health is inextricably tied to the social determinants of health, including educational attainment, poverty and income inequality, gender discrimination, racism, violence, and conflict [10]. People with low income, sexual minorities, racial and ethnic minorities or people with racialized identities, older people, people experiencing homelessness, and people with chronic health conditions are all particularly vulnerable to poor mental health and its sequelae [2]. Humanitarian emergencies and associated forced migration also increase the risk and prevalence of mental health conditions. An estimated 22% of people in conflict-related humanitarian crises experience a mood, anxiety, or psychotic disorder [11]. Sustainable global development is impossible without addressing population mental health needs through social interventions as well as interventions for direct care and treatment. Effective and affordable treatments have been developed but not distributed [12]. Globally, most people with mental illnesses receive no mental health care, and only a small percentage access quality, affordable, evidence-based care [13–15]. This is especially true of people who are socially marginalized, leading to substantial within-country inequities in access to care [16]. Within nearly all national health care budgets, mental health services are chronically under-funded, resulting in shortages of mental healthcare workers and services within countries [17, 18]. Typically, less than one percent of national health care budgets is earmarked for mental health–and that investment is commonly focused on centralized psychiatric services that meet only a small proportion of the overall need [17].

In line with the World Health Organization’s (WHO) strategic plan–the 13^th^ General Programme of Work (GPW 13) [19]–in 2019 WHO launched the *WHO Special Initiative for Mental Health*: *Universal Health Coverage for Mental Health* to advance mental health policies, advocacy, and human rights and to scale up access to quality and affordable care for people living with mental health conditions. With this historic initiative, WHO envisions a world in which all people achieve the highest standard of mental health and well-being. The goal is to expand access to mental health care to an additional 100 million people across multiple countries. To achieve this goal, two strategic actions will be taken. The first will be to develop and implement mental health policies and advance advocacy and human rights. The second will be to scale up effective mental health interventions in the community and in primary, secondary, and specialist care settings [4]. The mobilization of local champions and users of mental health services will be needed to achieve the first action, involving them in the design and implementation of laws, policies, plans, and services aligned to international human rights standards. Concurrently, advocacy must be carried out through both continued global and local campaigns so that mental health remains high on health, development, and humanitarian agendas. Equally important, laws, strategies and policies must be rooted in and operationalized based on international human rights standards, particularly the Convention on the Rights of Persons with Disabilities (CRPD) [20]. The second action will include increasing access to quality and affordable mental health services, leveraging available resources to integrate into available platforms and programs (e.g., primary care, refugee services, indigenous rights organizations), and developing priority service delivery to vulnerable populations across the life-course. Such services will need to be embedded in nationally strengthened mental health systems.

Actions and strategies will be carried out across distinct socio-cultural contexts through national health systems and non-governmental organizations. Effective planning and implementation will require a deep understanding of those systems and contexts. Situational assessment is therefore an important step: documenting, at the outset of the WHO Special Initiative for Mental Health, the specific conditions within each country that are potential facilitators of or potential barriers to successful mental health system expansion [19]. Relevant conditions include the social and environmental influences on mental health and wellbeing (e.g. quality education, income, exposure to gender or race-based discrimination, safe housing, neighborhood safety and cohesion), as well as health policies and plans, the prevalence of mental disorders by geographic area and demographic subgroup, the distribution of human and financial resources, the socio-cultural factors influencing attribution of mental health problems, as well as demand for and acceptability of mental health services.

Six countries were selected as ‘early-adopter’ countries for the WHO Special Initiative for Mental Health: Bangladesh, Jordan, Paraguay, the Philippines, Ukraine, and Zimbabwe. These countries–one in each of the six WHO regions–were chosen based on each government’s stated commitment to strengthening its mental health system. The objective of the baseline situational analysis was to use a rapid and comprehensive approach to assess the strength of the mental health systems in six countries. The results of the analysis will be used to inform national priority-setting and the development of country-specific theories of change at the outset of the WHO Special Initiative for Mental Health.

## Methods

We used a modified version of the PRIME situational analysis tool [21, 22] to assess the status of each country’s mental health system through a rapid and participatory process comprising review of existing literature and health system data, completion of facility checklists, and workshop discussions. The PRIME tool assesses six thematic areas: 1) socioeconomic and health context, 2) mental health policies and plans, 3) mental disorder prevalence and treatment coverage, 4) mental health services, 5) non-health sector/community-based services, and 6) monitoring and evaluation/health information systems. We expanded the PRIME tool to include multi-sector entry points for mental health promotion and services, a focus on equity and vulnerable populations, and stratification of relevant sociodemographic and health indicators across the life-course and by sex.

The assessment was carried out from December 2019 to March 2020, with most data collection finished by the end of January 2020. Six country teams–consisting of regional or country-specific WHO staff, consultants, and government representatives–worked closely with a six-member research team from the University of Washington (USA). The University of Washington (UW) team oriented each country team to the tool through a scheduled videoconference. Each country team subsequently met remotely on a weekly basis with the UW research team to guide data collection, including to identify and discuss data sources, review collected data, and verify data accuracy.

### Desk review

We conducted a multistep desk review to identify secondary data to populate the adapted PRIME tool. We searched PubMed and PsycInfo for peer-reviewed studies from each country, using broad search terms (mental health and country name). We searched the Scielo database for studies relevant to Paraguay. Most data on socioeconomic status, population health, policies, plans, and the mental health-related readiness of health and other sectors came from the following sources: mental health policies and plans, ministerial reports, and government documentation shared by country teams, World Bank reports, Demographic and Health Surveys, and the Global Health Observatory. All secondary data sources have been cited. Where possible, we accessed each country’s routine health information system to estimate counts of patients treated for priority mental health conditions in 2019 and counts of different types of mental health-related human resources and facilities. Essential drug lists in each country were reviewed for availability of psychotropic medications. Estimates of the prevalence of priority mental health conditions, stratified across the life course, were derived from a nationally representative mental health survey conducted in Bangladesh and from the 2019 Global Burden of Disease Study (GBD) for all other countries [23]. Priority mental health conditions in this desk review included: schizophrenia, bipolar disorder, major depressive disorder, epilepsy, alcohol abuse, drug abuse, and suicide deaths. Treatment coverage estimates were derived by dividing each country’s overall and age-specific counts of patients treated for each priority mental health condition by the respective 2019 GBD estimates of the number of people living with those conditions in 2019.

### Facility checklists

Country team members visited a convenience sample of health facilities in each country to document key indicators related to readiness to provide mental health services, including the availability of human resources for mental health, beds dedicated to mental health, psychiatric medications, and evidence-based psychotherapeutic interventions. Visits were conducted in person or by telephone. We adapted the WHO Service Availability and Readiness Assessment (SARA) instrument for this use [24]. We aimed to sample at least one specialist mental hospital, one psychiatric unit within a general hospital, and one primary care clinic per country. Facilities of each type were chosen for sampling based on the country team’s ability to travel to and access those facilities over the data collection period and in compliance with emerging COVID-19 restrictions in each country. Due to this convenience sample strategy, data from health facility checklists were not representative of each country’s mental health system nor its standard of care, but rather offered a snapshot of available services at a point in time for a sample of facilities. Findings from this data provided insight to differences in mental health policy and point in time service availability.

## Results

### Desk review—socioeconomic and health context

National demographic and population health characteristics for the participating countries are documented in Table 1. All six countries were classified as lower-middle or upper-middle income and were heterogeneous in population size and density, topography, history, culture, educational attainment, the availability of basic amenities, the rate of premature mortality, and the distribution of avertable morbidity. Each country faced unique challenges with implications for mental health service needs. Bangladesh and Zimbabwe both have large rural populations with limited access to socioeconomic resources. Notably, Jordan, with a total population of 9.5 million, is home to an estimated 1.2 million registered and unregistered refugees from the Syrian armed conflict [25, 26.] Bangladesh, with a population of 162.7 million [27], is losing arable land to severe climate events in high population-density regions and is home to over 800,000 Forcibly Displaced Myanmar Nationals [28, 29]. Ukraine has been suffering from a military conflict since 2014, and affected communities continue to experience hardship and human rights abuses [30]. Zimbabwe has a severe generalized HIV epidemic and, although public health measures have yielded progress in the reduction of new infections and mortality due to AIDS over the past decade, the psychosocial complications of the epidemic persist [31, 32]. In Paraguay, indigenous populations inhabiting remote regions have limited access to health services that are culturally competent and high quality [33, 34]. The Philippines is experiencing a rising sea level and 19–20 cyclones annually with 7–9 making landfall per year; families suffer the resulting adverse health consequences [35]. These social, political, and environmental conditions necessarily shaped each country’s approach to designing and delivering mental health services.

**Table 1:** National demographic and health characteristics.

	Bangladesh	Jordan	Paraguay	Philippines	Ukraine	Zimbabwe
**Demographic**						
Population	162,700,000[27]	9,531,712[36]	7,152,703[37]	106,651,922[38]	44,622,516[39]	14,030,368[40]
Under 14 years	30.4%[27]	34.5%[36]	23.4%[37]	31%[39]	16%[39]	44.4%[40]
Over 65 years	4.2%[27]	3.60%[36]	7.9%[37]	6%[39]	16%[39]	3.2%[40]
Rural population	62.6%[27]	9.0%[38]	32%[37]	53%[39]	30.5%[41]	67.8%[42]
Literacy	72.3%[27]	98.2%[43]	94.0%[44]	98%[44]	100%[45]	88.8%[44]
Languages	Bangla[46]	Arabic[47]	Spanish, Guaraní[48]	Filipino, English	Ukrainian, Russian[41]	Shona, Ndebele, English[42]
Ethnicities	Bengali (98.5%)[46]	Arab (98%)[47]	Mestizo (95%)[48]	Tagalog (28%), Cebuano (13.1%), Ilocano (9%), Bisaya (7.6%), Hiligaynon (7.5%), Bicol (6%), Waray (3.4%), Filipino-Chinese (2.5%)	Ukrainian (77.8%), Russian (17.3%), Belarusian (0.6%)[41]	African (99.4%)[42]
Religions	Muslim (89%), Hindu (10%)[46]	Muslim (97%)[47]	Roman Catholic (90%), Protestant (6%)[48]	Roman Catholic (81%), Protestant (8%), Muslim (6%)	Orthodox (66%), Greek Catholic (8–10%)[41]	Protestant (75%), Catholic (7%)[42]
GDP per capita	1,698 USD[49]	4,129 USD[49]	4,365 USD[49]	2,989 USD[50]	3,095 USD[51]	1,079 USD[49]
Income status[52]	Lower-middle income	Upper-middle income	Upper-middle income	Lower-middle income	Lower-middle income	Lower-middle income
Electricity (% homes)	32%[53]	100%[43]	98.4%[48]	91%[50]	99.8%[54]	33.7%[55]
Sanitation (% homes)	61%[53]	98%[43]	88.6%[48]	95%[50]	95.1%[54]	37%[55]
Water (% homes)	87%[53]	98%[43]	98.0%[48]	80%[50]	93.5%[54]	78.1%[55]
Education (% completed primary school)	60.3%[44]	95.9%[56]	84.2%[44]	72%[50]	98.7%[45]	88.4%[55]
**Health**						
Life expectancy at birth, years	72.2[39]	74.3[39]	74.5[37]	71[39]	71.8[39]	60.8[39]
Infant mortality (deaths/ 1,000 live births)	24[39]	17[39]	13.2[37]	21[57]	14[39]	39[39]
Maternal mortality (deaths/ 100,000 live births)	182[39]	29.8[39]	129[58]	121[57]	19[39]	462[55]
Leading causes of death	Stroke, Heart disease[59]	Heart disease, Stroke[43]	Heart disease, Road injuries[60]	Heart disease, Neoplasm[61]	Heart disease, Stroke[62]	Respiratory/TB, CVD, HIV[63]
Healthcare Access and Quality Index [64]	47.6	70	56.7	51.2	75	31.2
Universal Health Coverage Index [65]	33	73	66	53	49	34
HIV Seroprevalence	0.1%[27]	0.02%[66]	0.3%[67]	<0.1%[68]	1% (adults 15–49)[69]	13.8%[55]

### Mental health policies and plans

All six countries had stand-alone national mental health policies (Table 2). Policies express the vision, values, principles and objectives of a government and should include by a plan of action to advance population mental health[70]. The policies in each country emphasized deinstitutionalization and the decentralization of mental health services, all described approaches to integrate mental health services into primary health care, and all had specific provisions for the care of children and adolescents. Four countries had stand-alone plans for enacting their mental health policies; a new plan for Ukraine and a new policy and plan for Paraguay were in development at the time of data collection. Estimates of annual per capita mental health spending ranged from 0.08 USD in Bangladesh to 5.00 USD in Ukraine.

**Table 2:** Components of national mental health policies and plans.

	Bangladesh		Jordan		Paraguay		Philippines[71]		Ukraine		Zimbabwe	
	*Policy*	*Plan*	*Policy*	*Plan*	*Policy*	*Plan*	*Policy*	*Plan*	*Policy*	*Plan*	*Policy*	*Plan*
**Components**												
PHC integration						*n/s*				*n/s*		
Decentralization						*n/s*				*n/s*		
Hospital integration						*n/s*				*n/s*		
Maternal						*n/s*				*n/s*		
Child/adolescent						*n/s*				*n/s*		
HIV						*n/s*				*n/s*		
Alcohol/substance use						*n/s*				*n/s*		
Epilepsy						*n/s*				*n/s*		
Dementia						*n/s*				*n/s*		
Promotion/prevention						*n/s*				*n/s*		
Suicide						*n/s*				*n/s*		
**Equity**												
Gender						*n/s*				*n/s*		
Age/life course						*n/s*				*n/s*		
Rural/urban						*n/s*				*n/s*		
Socio-economic status						*n/s*				*n/s*		
Vulnerable populations						*n/s*				*n/s*		
**Public Spending on Mental Health** *(USD/capita)*	0.08 USD		*n/s*		0.37 USD		0.47 USD		5.00 USD		0.13 USD	
**Legend**												
Present		Absent		Not specified/no data			*n/s*					

### Mental disorder prevalence and treatment coverage

Table 3 shows overall and population-specific prevalence estimates of selected mental disorders, as well as estimates of overall and population-specific treatment coverage where available. Schizophrenia, epilepsy, and drug abuse prevalence estimates were relatively consistent across countries. Paraguay had the highest estimated prevalence of bipolar disorder (1.2%). Bangladesh had the highest estimated prevalence of major depressive disorder (6.7%). Ukraine had the highest estimated prevalence of alcohol use disorders (3.0%) as well as the highest estimated death rate from suicide (31.1 per 100,000 population). Men were estimated to be at disproportionate risk of alcohol abuse and suicide in all countries, while women were at disproportionate risk of major depressive disorder. Treatment coverage estimates were available in Paraguay, Philippines, and Ukraine. In Paraguay, the Philippines, and Ukraine, the treatment coverage for schizophrenia was substantially higher than for most other conditions (50.0%, 19.0%, and 9.4% treatment coverage, respectively). Men were estimated to have greater access to treatment for severe mental disorders, epilepsy, and drug abuse in Ukraine, and for epilepsy and drug abuse in Paraguay.

**Table 3:** Estimated prevalence and treatment coverage of selected mental disorders.

	Bangladesh		Jordan		Paraguay		Philippines		Ukraine		Zimbabwe	
	*NHMS Prevalence[72]*	*Treated*	*GBD Prevalence[73]*	*Treated*	*GBD Prevalence[73]*	*Treated*	*GBD Prevalence[73]*	*Treated*	*GBD Prevalence[73]*	*Treated*	*GBD Prevalence[73]*	*Treated*
**Schizophrenia**												
Overall	1.0%	--	0.3%	--	0.3%	50.0%	0.3%	19.0%	0.4%	9.4%**	0.2%	--
Female	1.1%	--	0.2%	--	0.3%	50.0%	0.3%	--	0.3%	8.1%**	0.2%	--
Male	0.9%	--	0.3%	--	0.3%	50.0%	0.3%	--	0.4%	11.5%**	0.2%	--
15–19 years	--	--	0.1%	--	0.1%	--	0.1%	--	0.1%	--	0.1%	--
20–29 years	--	--	0.3%	--	0.3%	51.0%	0.4%	--	0.3%	6.7%**	0.2%	--
70+ years	--	--	0.3%	--	0.3%	70.0%	0.3%	--	0.4%	6.9%**	0.2%	--
**Bipolar Disorder**												
Overall	0.5%	--	0.8%	--	1.2%	3.0%	0.3%	5.0%	0.6%		0.5%	--
Female	0.3%	--	0.9%	--	1.2%	4.0%	0.3%	--	0.7%		0.5%	--
Male	0.7%	--	0.8%	--	1.1%	2.0%	0.3%	--	0.6%		0.5%	--
15–19 years	--	--	1.1%	--	1.6%	--	0.3%	--	0.6%		0.7%	
20–29 years	--	--	1.2%	--	1.8%	2.0%	0.5%	--	0.8%		0.8%	--
70+ years	--	--	0.8%	--	1.2%	5.0%	0.3%	--	0.7%		0.5%	--
**Major Depressive Disorder**												
Overall	6.7%	--	3.2%	--	3.0%	8.0%	1.4%	1.0%	3.9%		1.6%	--
Female	7.9%	--	4.2%	--	4.4%	9.0%	1.6%	--	4.8%		1.9%	--
Male	5.4%	--	2.2%	--	1.5%	6.0%	1.2%	--	2.9%		1.3%	--
15–19 years	--		3.5%	--	3.9%	--	1.6%	--	1.9%		1.4%	--
20–29 years	--	--	4.1%	--	3.8%	5.0%	2.0%	--	3.1%		2.0%	--
70+ years	--	--	3.2%	--	2.9%	12.0%	1.4%	--	3.6%		1.5%	--
**Epilepsy**												
Overall	0.3%	--	0.3%	--	0.4%	22.0%	0.3%	1.0%	0.3%	38.0%	0.4%	--
Female	0.1%	--	0.3%	--	0.4%	19.0%	0.3%	--	0.3%	33.0%	0.3%	--
Male	0.9%	--	0.3%	--	0.5%	25.0%	0.3%	--	0.3%	43.0%	0.4%	--
15–19 years	--		0.4%	--	0.5%	--	0.3%	--	0.3%	--	0.4%	--
20–29 years	--	--	0.3%	--	0.5%	0.0%	0.2%	--	0.3%	--	0.4%	--
70+ years	--	--	0.3%	--	0.4%	12.0%	0.2%	--	0.3%	--	0.3%	--
**Alcohol Abuse**												
Overall	1.5%¶	--	0.4%	--	2.8%	1.0%	0.8%	1.0%	3.0%	21%§	1.5%	--
Female	1.0%¶	--	0.3%	--	1.4%	1.0%	0.3%	--	1.7%	20%§	0.5%	--
Male	1.9%¶	--	0.6%	--	4.2%	1.0%	1.3%	--	4.6%	17%§	2.6%	--
15–19 years	0.6%¶	--	0.3%	--	1.3%	--	0.4%	--	0.8%	--	0.7%	
20–29 years	2.5%¶	--	0.8%	--	4.2%	0.0%	1.2%	--	3.0%	--	2.7%	--
70+ years	0.8%¶	--	0.4%	--	2.8%	3.0%	0.8%	--	3.2%	--	1.5%	--
**Drug Abuse**												
Overall	0.5%	--	0.5%	--	0.8%	11.0%	0.6%	--	0.8%	35.0%	0.6%	--
Female	0.1%	--	0.4%	--	0.9%	5.0%	0.5%	--	0.4%	13.0%	0.5%	--
Male	0.9%	--	0.6%	--	0.7%	15.0%	0.7%	--	1.3%	38.0%	0.8%	--
15–19 years	--		0.6%	--	1.0%	--	0.9%	--	1.2%	--	0.9%	--
20–29 years	--	--	1.2%	--	2.3%	10.0%	1.6%	--	2.6%	32%†	1.5%	--
70+ years	--	--	0.5%	--	0.8%	9.0%	0.6%	--	0.9%	9%‡	0.6%	--
**Suicide Deaths** *												
Overall	6.0¶	--	2.2	--	6.2	--	4.1	--	31.1	--	16.0	--
Female	6.0¶	--	0.9	--	3.0	--	1.5	--	9.0	--	8.9	--
Male	5.9¶	--	3.4	--	9.3	--	6.5	--	56.9	--	23.7	--
15–19 years	5.3¶	--	1.5	--	8.7	--	3.6	--	20.6	--	14.3	--
20–29 years	9.7¶	--	3.1	--	10.0	--	7.0	--	39.3	--	16.5	--
70+ years	11.2¶	--	2.1	--	6.1	--	4.0	--	30.7	--	14.8	--

*Rate per 100,000 population

**Treated prevalence reported for schizophrenia, bipolar disorder, and major depressive disorder

† treated age group includes 18–35 years while prevalence age group includes 20–34 years

‡ treated age group includes 60+ while prevalence age group includes 70+

§ Alcohol use disorder data comes from the WHO Global Alcohol Report for Ukraine[74]

¶ Data from GBD 2019*[73]*

### Mental health services

**Fig 1:**
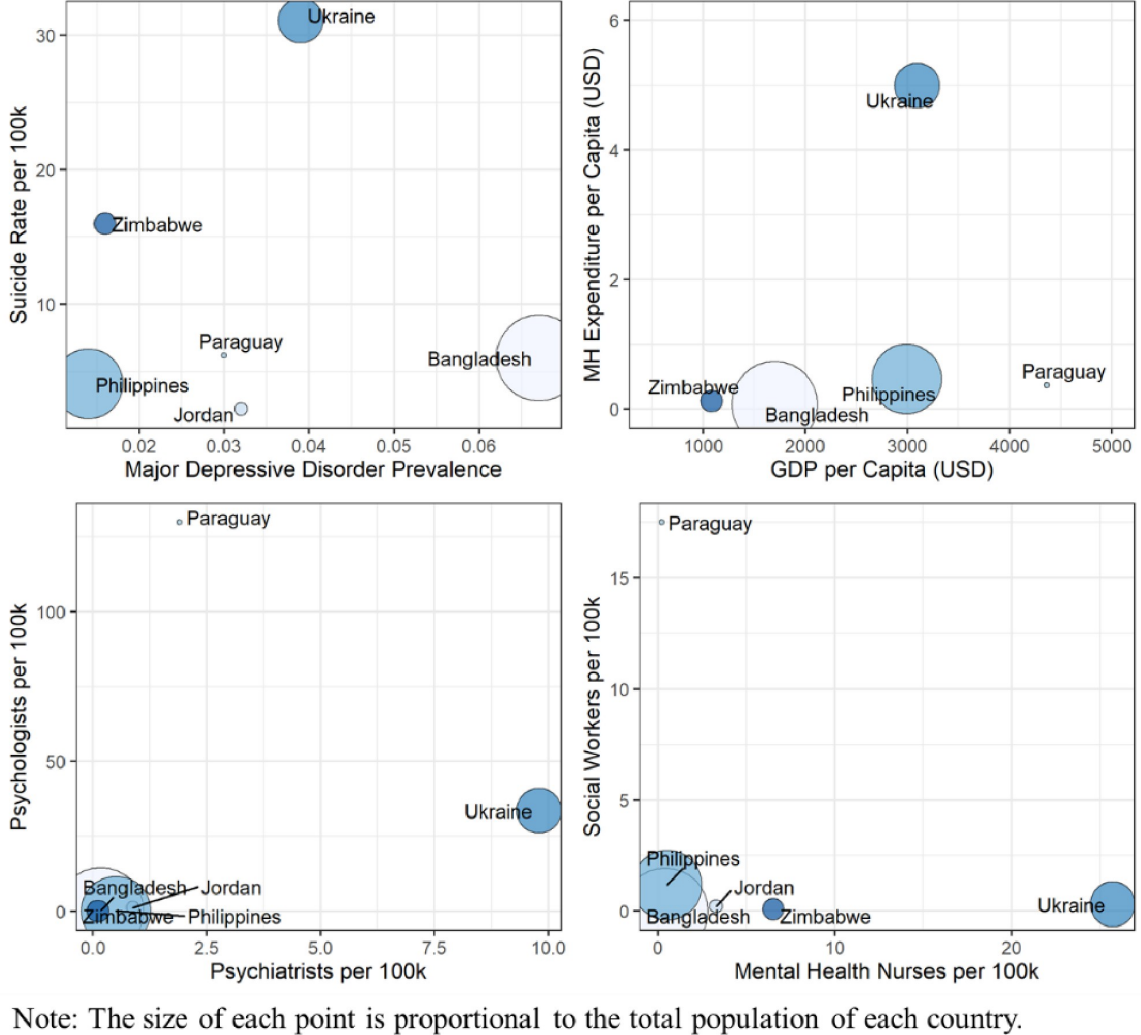
Comparisons of country mental health characteristics.

The distribution of human resources and facilities dedicated to mental health care differed across the six countries (Table 4). Resources and facilities reported were primarily for the public sector only. Zimbabwe had few psychiatrists (18, or 0.01 per 100,000 population) and a relatively large number of psychiatric nurses (917, or 6.5 per 100,000). Bangladesh had 250 psychiatrists (0.2 per 100,000 people) and 565 psychologists for its population (0.3 per 100,000). Ukraine had nearly 10 psychiatrists and 12 neurologists per 100,000 people. Paraguay had a relatively large number of psychologists (9,143, or 129.8 per 100,000) and between 1.5 and 2 psychiatrists per 100,000 with the public and private sectors combined. Social workers in the health system were more plentiful in the Philippines and Paraguay. Table 4 also shows the considerable differences in available inpatient beds and outpatient services across the six countries. Apart from Ukraine, forensic units and alcohol/drug specialty facilities were rare; although this finding may not be inclusive of privately run alcohol/drug specialty facilities for which there is limited regulation. All countries lacked child/adolescent specialty facilities. Fig 1 displays the country data in relation to these dimensions.

**Table 4:** Human resources and health facilities for mental health.

	Bangladesh‡‡		Jordan‡‡		Paraguay[75]		Philippines‡‡		Ukraine‡‡		Zimbabwe‡‡	
	*#*	*/100k*	*#*	*/100k*	*#*	*/100k*	*#*	*/100k*	*#*	*/100k*	*#*	*/100k*
**Generalist**												
Doctor	20,914	12.9	20,160	211.5	20,404	285.3	40775*	38.2	33,730	75.9	2,245	16.0
Nurse	27,432	16.9	22,540	236.5	35,458	495.7	90308*	84.7	253,780	568.7	10,102	72.0
Pharmacist	--	--	13,554	142.2	3,133	43.8	--	--	521††	1.2	--	--
Feldsher *(physicia* *n assistant)*	--	--	--	--	--	--	--	--	30,232	67.8	--	--
**Specialist**												
Neurologist	225	0.1	--	--	63	0.9	483	0.5	5,522[76]	12.4	4	0.03
Psychiatrist	250†	0.2	87	0.9	136	1.9	548	0.5	4,363	9.8	18	0.1
Psychologist	565	0.3	124	1.3[17]	9,143	127.8	133	0.1	15,061[77]	33.6	6	0.04
Psychiatric nurse	700	0.4	315	3.3[17]	12	0.2	516	0.5	11,477	25.7	917	6.5
MH social worker	3	0	19	0.2[17]	1,230	17.2	1,241	1.2	128	0.3	13	0.09
**Inpatient**												
Mental hospital												
*Facilities*	2[17]	0.001[17]	5	0.05	2	0.03	4	0.004	169‡	0.4	2	0.01
*Beds*	700[17]	0.4[17]	560	5.9	359	5.0	4,373	4.1[17]	27,447	61.5	1,614	11.5
General hospital psychiatric												
*Facilities*	56	0.03	3	0.03	2	0.03	46	0.04	41[78]	0.09	2	0.01
*Beds*	504	0.3	39	0.4	14	0.2	--	--	1,048	2.3	142	1.0
Forensic unit												
*Facilities*	1	0.0006	1	0.01	1	0.01	--	--	53[78]	0.1	2	0.01
*Beds*	16	0.01	140	1.5	42	0.6	--	--	--	--	195	1.4
Residential care												
*Facilities*	72	0.04	--	--	6	0.08	63	0.06	167[79]	0.4	10	0.07
*Beds*	3,645	2.2	--	--	63	0.9			30,889[79]	69.2	72	0.5
Child/adolescent												
*Facilities*	2	0.001	0	0	3	0.04	--	--	60[78]	0.1	--	--
*Beds*	33	0.02	0	0	--	--	--	--	1,296	2.9	--	--
Alcohol/drug												
*Facilities*	5§	0.003	2	0.02	1	0.01	--	--	110[78]	0.2	1	0.007
*Beds*	--	--	47‖	0.5	--	--	--	--	3,406	7.6	--	--
**Outpatient**												
Hospital	69	0.04	30¶	0.3	59	0.8	29	0.03	626**	1.4	7	0.05
Community-based	--	--	83¶	0.9	32	0.4	1,362	1.3	--	--	--	--
Child /adolescent	20	0.01	3	0.03	3	0.04	--	--	561[78]	1.3	2	0.01

*Number in institutions[80]

†The Bangladesh Association of Psychiatrists reports 250 psychiatrists

‡All facilities providing any inpatient care, some provide other types of care in addition (i.e. outpatient)

§ Government-owned facilities

‖ Number of beds unknown at one of the two facilities

¶ RMS not included

**All facilities providing any outpatient care, some provide other types of care in addition (i.e. inpatient)

††521 pharmacists work in hospitals (there are 111 pharmacies as departments of hospitals), and in Ukraine there are 20,600 pharmacies (mostly private) with unknown number of staff[81]

‡‡All numbers provided by data filled out by country teams in the situational analysis tool unless otherwise cited

### Facility checklists

Across five of the six countries, team members visited 27 health facilities during the initial assessment period (Table 5). Due to time and resource constraints, the team in Paraguay was not able to complete facility checklists. The data provided snapshots of service delivery conditions in specific institutions but did not characterize all treatment settings. Major, national-level referral facilities for mental health generally had large complements of psychiatrists and mental health nurses; psychologists were rare. The facilities had comprehensive access to essential psychiatric medications and typically had clinical staff who offered cognitive behavioral therapy (CBT), which is an evidence-based psychotherapeutic intervention. Fewer services were available at secondary and primary care facilities; these generally had few or no psychotropic medications or psychological interventions available. The exceptions were facilities that were implementing the WHO Mental Health Gap Access Program (mhGAP) [82] (e.g., Ukraine, Philippines) or affiliated with civil society organizations providing psychological services (e.g., Friendship Bench in Zimbabwe).

**Table 5:** Characteristics of selected health facilities*.

Description of Selected Health Facility	Psychiatrists	Psychiatric Nurses	Psychologists	Mental Health Beds	Psychiatric Medications Available	Psychiatric Interventions Available
**Bangladesh**						
National referral mental hospital with inpatient and outpatient services. Teaching facility. MoHFW. Urban.	28	250†	1	200	Comprehensive, available‡	PST, BAT, supportive counselling, CBT, IPT, brief alcohol interventions, MET, PP, family support
National referral hospital with inpatient psychiatric ward and Outpatient mental health services. MoHFW. Urban.	2	3	16	22	Partially comprehensive, inadequate supply§	PST, BAT, supportive counselling, CBT, IPT
Health centre/clinic. Outpatient, only. MoHFW. Rural.	0	0	0	0	Diazepam, only	Supportive counselling
Jordan						
Major mental hospital. Focus on medication management. MoH. Urban.	17	4	7	205	Comprehensive, available‡	None
Women’s psychiatric unit in general hospital. Focus on medication; few psychosocial services. Few patients. MoH. Urban.	3	2	1	12	Comprehensive, available‡	Uncertified: supportive counselling, CBT, family support
Psychiatric unit in medical centre. Inpatient service. Army. Urban.	13	1	4	38	Comprehensive, available‡	PST, supportive counselling, CBT, MET, family support
Outpatient clinic. Few referrals to psychologist. MoH. Urban.	2	*n/s*	1	0	Comprehensive, available‡	PST
Outpatient clinic. Serving refugees. NGO. Urban.	1‖	0	1	0	Comprehensive, available‡	Case management, PST, BAT, supportive counselling, CBT, IPT
Primary care clinic. Mental health integrated through mhGAP and family medicine physician. MoH. Rural.	0	0	0	0	None	Psychoeducation
**Philippines**						
Major mental hospital and research training centre. MoH. Urban.	68	409	16	4200	Comprehensive, available‡	PST, supportive counselling, CBT, IPT, MET, PP, family support
Regional mental hospital. Rural. MoH.	6	113	1	500	Comprehensive, available‡	Supportive counselling, CBT, family support
Drug recovery clinic. No funding for medication. MoH. Urban.	1	1	1	0	None	PST, supportive counselling, CBT, IPT, brief alcohol, MET, PP, family support
Municipal health office providing some mental health services and outreach through community health workers. MoH. Urban.	0	0	0	0	Risperidone and phenytoin	Supportive counselling, brief intervention for alcohol
Municipal health office providing follow-up to patients referred from specialist care. MoH. Rural.	0	0	0	0	Risperidone	None (referrals only)
**Ukraine**						
Regional psychiatric hospital. Pharmacological and psychosocial treatment. Municipal. Urban and Rural.	26	131	5	275	Comprehensive, available‡	CBT, brief alcohol intervention, MET
National forensic psychiatric facility providing compulsory treatment. Municipal. Urban.	6	0	2	120	Comprehensive, available‡	PST, BAT, supportive counselling, CBT, PP, art therapy
District-level general hospital with inpatient and outpatient mental health services. Municipal. Urban and Rural.	3	8	0	17	Comprehensive, available‡	Supportive counselling, brief alcohol intervention, art therapy
Primary health care centre implementing mhGAP. Municipal. Urban and Rural.	0	0	0	0	Comprehensive, available‡	mhGAP interventions
Primary health care centre. Some mental health care provided, but not mhGAP. Municipal. Urban.	0	0	0	0	Comprehensive, available‡	Supportive counselling
**Zimbabwe**						
National mental hospital. MoH. Urban.	4	19	1	100	Comprehensive but low supply‡	PST, BAT, supportive counselling, CBT, IPT, brief alcohol interventions, MET, family support
National forensic hospital providing compulsory services. Urban. MoH.	1	8	0	50¶	Comprehensive, available‡	PST, supportive counselling, CBT, IPT, brief alcohol interventions, MET
Provincial hospital with inpatient psychiatric ward. MoH. Urban.	1**	0	3	14	Comprehensive but low supply‡	None
Primary health care centre implementing Friendship Bench. MoH. Urban.	0	0	1	0	Comprehensive but low supply‡	PST, supportive counselling, brief alcohol intervention, family support

*This table reflects data collected from Health Facility Checklists completed in person in early 2020. The data is not exhaustive representation of care or standards of care.

†Degree nurses who work in the psychiatric hospital

‡Meets or exceeds criteria defined by World Health Organization Model List of Essential Medicines, 2019

§Does not meet criteria defined by WHO Model List of Essential Medicines, 2019

‖ Psychiatrist works 3 days in the clinic and supervises PHC clinics the other 2 days/ week

¶Many sleep on floor

**Part-time

Abbreviations. MoHFW: Ministry of Health & Family Welfare, MoH: Ministry of Health. NGO: Non-governmental organization. PT: Part-time. PP: Positive psychotherapy. BAT: Behavioral activation therapy. CBT: cognitive behavioural therapy. PST: problem solving therapy. MET: motivation enhancement therapy. IPT: interpersonal therapy. *n/s*: not specific/no data

### Non-health sector/community-based services

A diverse range of non-health sector mental health entry points and services were identified (Table 6). Members of the clergy and other religious or traditional healers served as important sources of psychosocial support for mental health problems in Bangladesh, Jordan, Ukraine, and Zimbabwe. In Ukraine, clergy reported praying, listening to confession, talking through problems, and sometimes suggesting meditation in response to people with symptoms of anxiety, depression or problem alcohol use [83]. Mental health promotion activities were carried out through health education and advocacy at the community level, the workplace, treatment centers, and schools. Ukraine and the Philippines reported countrywide integration of mental health services and promotion into their public school systems [84–87]. Ukraine had engaged a cadre of school-based mental health workers. The Philippines public education system provided pre-school education to more than 12 million children, and its K-12 health curriculum included competencies in social and emotional health and substance use prevention education. In Jordan, Bangladesh, and Ukraine, non-governmental organizations provided substantial mental health and psychosocial support (MHPSS) services specifically for refugees and other displaced people or for people affected by conflict and humanitarian crisis. Social welfare and refugee services in the Philippines disseminated MHPSS training (e.g., in psychological first aid) to prepare responders for disasters. In Ukraine, the social welfare sector provided services for people with severe mental disorders through residential care facilities.

**Table 6:** Non-health sector mental health capacity.

	Bangladesh	Jordan	Paraguay	Philippines	Ukraine	Zimbabwe
**Community**	Traditional healers serve an estimated 40% of persons with MH conditions, religious healers also prominent in mental health care delivery	Physical and mental illness is sought through religious healing.[88, 89]	Sheltered housing exists across the country, mainly in Asunción and the Central Department; telemedicine pilot for delivering mental health care; mobile outreach unit for mental health care delivery	National Anti-Poverty Commission (NAPC) focuses on food, shelter, water, healthcare, work, education, social protection, peace, and environment.[90]	Community mental health care is a relatively new concept in the country and continues to evolve as part of decentralization reform. Mental health care is highly stigmatized and awareness is still low in the communities. Self-medication and reluctance to seek help are very common.[83]	Faith healers practice throughout the country.[91–93]
**Education**	Lack of special education programs, school counsellors, or MH literacy for teachers in schools; a few private schools have specialty programs for students with developmental disabilities and other mental disorders	Some targeted education within refugee services.[94] Efforts are ongoing to scale up the School Mental Health Package.[95]	Ministry of Education working in schools to identify youth with mental health concerns	The public education system provides preschool education to more than 12 Million children; mental health topics covered in K-12 and special education programs.[86, 87] The Philippines has integrated MH services and promotion in schools under the new Mental Health Act.[96]	The education sector in Ukraine engages in mental health activities through school-based mental health workers, launching new programs, and incorporating mental health literacy into teacher training and school-based activities. [84, 85]	Policy stipulates presence of guidance counsellors in all schools.
**Social Welfare**	Department has special program and monthly allowance for persons with disability, including MH. Neurodevelopmental Disability Protection Trustee Board works for children with NDD’s. Department sponsors correction centers for juvenile delinquency.	35 NGOs deliver MHPSS services, programs, and activities and are coordinated through the MHPSS Working Group.[94]	Economic support provided to people with diagnoses of psychosocial disabilities (among other disabilities); Institute of Social Welfare administers homes for the elderly and deinstitutionalized people from the Psychiatric Hospital.	The Social Reform and Poverty Alleviation Program has specific/tailored projects for certain population groups—artisanal fisherfolk, children/youths/students, cooperatives, farmers and landless rural workers, indigenous peoples and cultural communities, persons with disabilities, urban poor, senior citizens, women, formal labor and migrant workers.[90, 97]	Social welfare sector is independent from health and does not offer psychosocial support for people with mental health disorders in community. Around 160 residential facilities (internats) for people with severe mental disabilities are under the social welfare sector, and are often associated with human rights violations. The government aims to shift focus of social welfare sector to more recovery based and patient centered approach.[98]	Queen of Peace Rehabilitation and Crisis Centre provides essential re-integration services to discharged individuals.
**Justice**	The criminal justice system offers programming geared towards prevention and treatment of substance abuse.	Mental health and psychosocial services need to be scaled up. Within the juvenile criminal justice system, there are proposed actions that emphasize the integration of psychosocial interventions at various stages.[99, 100]	Ministry of Justice provides psychiatric medicine to people in prison with mental health conditions.	Juvenile Justice and Welfare Act of 2006;[101] Bureau of Jail Management and Penology (BJMP) on mental health campaign—training for psychometricians.[102]	In 2018, there were 77 psychiatrists providing psychiatric care to people in prisons and detention facilities. Mental health care for prisoners does not follow the WHO standards[103]	Not specified

### Cross-cutting themes

Several common themes emerged from the situational assessment process, reflecting goals and challenges shared by each country. These are each discussed below.

#### Decentralization and deinstitutionalization

All countries indicated plans to shift from a focus on specialist facilities offering institutionalization located in urban centers to community-based care and non-specialized providers dispersed across smaller urban centers and rural communities; although the extent this was reflected in concrete community, primary and non-specialist services was limited. With this shift came the possible redistribution of existing specialists to underserved areas in the future. Several countries had also invested in mental health training for generalist nurses and doctors. The Philippines shifted decision-making for most healthcare spending from the central level to local government units. In 2017, the government of Ukraine passed the Concept of Mental Health Development in Ukraine (2018 to 2030), with planned efforts to improve accessibility of mental health services through deinstitutionalization, to develop out-of-hospital forms of assistance, and to integrate mental health services into primary care. In response to the need for expanded access to quality mental health care in the community that promotes human rights, Paraguay’s health system implemented Unidades Moviles de Salud Mental (mobile mental health units) in 2004, whose expansion continues. By 2018, these mobile units were delivering care to people in more remote areas of the country [104]. For people with severe mental illnesses, hogares sustitutos, residential care facilities in the community, provided an alternative to psychiatric hospitalization that permits greater social integration for a relatively small number of people [104]. Critically, in several countries, although deinstitutionalization and decentralization were in process, a gap remained in the availability of mental health specialists to offer care at secondary levels and to train, supervise and support mental health care providers at primary and community levels. Related to this, the provision of mental health services in general hospitals (i.e., secondary care) remained a need in most of the countries.

#### Integration of mental health care into other services

All countries were seeking to strengthen mental health services at the primary care level to facilitate decentralization, to de-stigmatize services, and to expand service access. A common step taken across countries was to pilot the implementation of mhGAP trainings to selected primary care facilities. In Bangladesh, more than 300 physicians, psychologists and counsellors working with governmental and nongovernmental organizations in Cox’s Bazar district had participated in these trainings since November 2017, held in response to the humanitarian crisis [105]. Following Typhoon Yolanda in 2014, the Philippines trained primary care physicians on mhGAP with support from WHO. However, post-training uptake and maintenance of the mhGAP program was limited; few countries had systems in place for ongoing supervision of primary care providers in mhGAP. Paraguay began mhGAP trainings in Asuncion-Capital and Central in 2019 and expanded access to 585 participants using a virtual platform by 2020. The country laid the groundwork for mhGAP integration by increasing the number of family health units, which now total 804 and could serve as platforms for delivering community-oriented mental health care [106]. Zimbabwe is home to an innovative program called the Friendship Bench [107], which has received worldwide attention and is supported by international funding. The Friendship Bench program demonstrated effectiveness treating people with depressive symptoms through primary care-based lay counsellors trained in problem-solving therapy.

#### Financial and human resources for mental health

Most countries cited limited financial and human resources for mental health as a major impediment to further strengthening their systems of care. There were an insufficient number of specialist providers available in most countries to offer evidence-based services to patients or to train and supervise generalist providers working in decentralized health care settings. The limited human and financial resources tended to be inequitably distributed across geography or population groups within each country, given the centralized structure of service delivery and continued reliance on psychiatric hospitals. In Ukraine, almost all mental health system resources were spent on specialist psychiatric hospitals or residential care facilities. Though the Philippines had a strong medical education system and produced many specialist trainees, the country also lost workers to high-income countries [108]. Almost all remaining specialists worked in Manila, leaving many provinces in the Philippines with no mental health specialists. Zimbabwe had similarly lost many locally trained professionals due to economic and political instability, and the remaining psychiatrists and psychologists lived exclusively in major urban areas. Notably, though Zimbabwe had many trained psychiatric nurses, the majority of these had transitioned to HIV-related care, which was well funded by international donors. In Paraguay, most specialized mental health professionals were concentrated in the capital city, Asuncion, although a recently launched pilot project was sending these providers to other regions and had increased their numbers in the interior of the country.

## Discussion

We conducted a rapid assessment of the needs and strengths of mental health systems in Bangladesh, Jordan, Paraguay, the Philippines, Ukraine, and Zimbabwe, as part of the launch of the WHO Special Initiative for Mental Health: Universal Health Coverage for Mental Health. We used an adapted version of the PRIME situational analysis tool [21, 22], including a more comprehensive range of community-level and multi-sector service platforms, a focus on vulnerable populations, and stratification of several indicators by sex and across the life-course. Though countries were heterogeneous in their mental health resources and needs, several common themes emerged. Most countries were dealing with national crises that had serious implications for population mental health, and none had adequate mental health services to meet even a fraction of their need. All were aiming to decentralize and deinstitutionalize their mental health services, to integrate mental health care into primary health care, and to devote more financial and human resources to mental health systems.

Several of these findings are relevant for comparable countries also seeking to prioritize mental health as part of efforts to expand universal health coverage. Though sampled countries have made progress toward deinstitutionalization and decentralization, the lack of strong mental health services at primary care facilities and at the community level was a consistent finding. Globally, 80% of public spending on mental health services in LMICs is invested in mental hospitals [17]. For example, a recent analysis in South Africa showed that 86% of mental healthcare expenditures paid for inpatient services, nearly half of which went to psychiatric hospitals [109]. Increased financial investment in and political commitment towards community- and primary care-based services is warranted, as is the exploration of appropriate task-sharing of some mental health-related activities to generalist and community-based providers [110]. Importantly, though within-country inequities in need for and access to mental health services are likely substantial, we were generally unable to find routine data that were sufficient to estimate and understand these inequities in detail. We identified policy statements or programmatic initiatives in each country targeting populations that were vulnerable because of forcible displacement or social marginalization, though it was usually not possible to describe their reach or effectiveness.

At the end of January 2020, representatives of mental health systems from each of the six countries met with representatives from the Situational Analysis Implementation Team and WHO mental health leadership at the WHO headquarters in Geneva for a multi-country meeting to commence the WHO Special Initiative for Mental Health work. Representatives included people with lived experience of mental health conditions, mental health advocates, Ministry of Health officials, heads of psychiatric hospitals, and mental health sector administrators. At this meeting, we reviewed the findings of the rapid assessment and held preliminary discussions on priority-setting and program design. Each country team chose five key priorities that would influence their country specific WHO Special Initiative for Mental Health work plans. These priorities tended to align with the common themes identified in the formal assessment, though several priorities were set via group discussions that were influenced by people with lived experiences of mental health conditions and other experts. For example, primary care integration and health workforce development were key priorities that emerged from the assessment process. However, improving the quality of care, which had not come to light in the assessment, was recognized and assigned higher priority. Strengthening the inclusion of civil society members in service design and in monitoring adherence to human rights standards arose as an additional priority, aligning with the initiative’s emphasis on human rights. WHO identifies engagement of service users and families as a key component of the development and reform of mental health services [17]. Each country acknowledged the need to increase the participation of persons with lived experience in decision making, to foster greater coordination among advocacy groups, and to establish formal funding mechanisms to support these groups. Other commonly identified priorities included strengthening awareness and knowledge about mental health and improving routine health information systems. These activities will feature in country plans only where they support priority areas for the WHO Special Initiative for Mental Health. Scaling up effective community-based mental health services, building governance capacity, coordinating mental health planning and program implementation across multiple sectors and reviewing and implementing national policies and plans for mental health were all priorities for countries and for the WHO Special Initiative for Mental Health. Assessment findings also contributed to refining or engaging more targeted interventions in country plans. For example, Zimbabwe’s assessment that showed multiple psychiatric forensic services warranted service-planning that intersected with the justice system, while prevalence data from Paraguay showed a clear need for youth oriented mental health and substance use services.

This difference in priorities emerging from the assessment and from in-person stakeholder discussions reflected the reliance of the assessment process on routinely available data and a small number of facility visits. Priorities like health workforce development could be deduced from provider counts and treatment coverage estimates, whereas the need to strengthen care quality or build governance for multi-sector coordination had to be inferred or distilled from discussions with in-country stakeholders. That is, routine data did not capture the attitudes and knowledge of key system actors; these are critical to understanding the context and identifying accessible “levers” that could be pulled to effect change [111]. A mixed method approach to situational analyses–combining quantitative and qualitative data to triangulate findings, deepen understanding, and capture the full complexity of health systems within cultural contexts–would mitigate this limitation [112].

Several additional limitations to our approach must be acknowledged. First, routine data were inconsistently available and often of poor-quality (e.g., unreliable, biased, or unable to be disaggregated). For example, only half of the countries had health information systems capable of estimating counts of patients treated for specific mental health conditions, and only one country, Bangladesh, had conducted a nationally representative population mental health survey. Ukraine successfully integrated modules on depression and suicidal behavior into its WHO STEPwise approach to surveillance (STEPS) survey system [113]. Jordan did not have available data on per capita spending for mental health. Countries rarely had any information on human resources and facilities in the private sector, therefore nearly all data reflects public and NGO sectors. Additionally, available data on human resources and health facilities was often limited to public sector facilities, which limited our ability to generalize results to the entire mental health care system of countries. Moreover, little information on non-health sector engagement in the mental health system was available from any of the six countries. Although strengthening national mental health information systems in low- and middle-income countries has been identified as a key priority in global mental health, many systems remain under-resourced and under-developed [114]. Nonetheless, we were able to combine counts of treated patients in three countries with imputed prevalence estimates from the Global Burden of Disease (GBD) study to calculate rough treatment coverage estimates, highlighting the potential usefulness of combining available data in novel ways to assess system strength. Simultaneously building routine health information systems to collect reliable data while expanding the delivery of much needed mental health and social services may offer opportunities to engage vulnerable population subgroups in care—such as children, elderly citizens, refugees, persons living in poverty, prisoners, and families adversely affected by climate change—and to better understand the nuances of the unmet need for mental health services within each country.

The country assessments had to be both rapid and broad, as they were conducted on a short timeline and intended to start discussions for planning and design of a program of work for each country. We were therefore not able to generate detailed, targeted assessments of specific system weaknesses or opportunities, nor was it feasible to cover all possible issues related to mental health. Some important political and legislative concerns (e.g., the criminalization of suicidality) were not reviewed. On the other hand, our approach was efficient, lasting only six weeks, and despite their limitations the assessments facilitated robust discussion around priority-setting and program design by national system stakeholders. Many details from country assessments have not been presented in this manuscript to minimize its length; detailed reports are available online [4].

In summary, we report the results of a situational analysis of mental health systems in Bangladesh, Jordan, Paraguay, Philippines, Ukraine, and Zimbabwe, which was conducted as an initial step in launching the WHO Special Initiative for Mental Health: Universal Health Coverage for Mental Health. Despite the rapid nature of the assessment and reliance on routine, publicly available data, the assessment helped to facilitate priority-setting among country stakeholders. Information from our review of quantitative data was enhanced by the guidance, hard work, and perspectives that country team members contributed during the six-week assessment process, and by information obtained through facility visits. Planning for the WHO Special Initiative for Mental Health was enhanced through the forum of collaborative activities that were implemented by WHO during meetings convened in Geneva. Moving forward, WHO is using these assessment findings to facilitate design workshops with stakeholders in each country to formalize priorities and detailed plans for building stronger mental health systems. Components of the assessment will be repeated or expanded in each country over the course of the WHO Special Initiative for Mental Health as part of country-specific monitoring and evaluation procedures with the goal of documenting progress and estimating impact.
